# Antibiotic resistance: A cross-sectional study on the characteristics, knowledge, attitudes, and practices of dairy farmers’ cooperative in North Cianjur (KPSCU), Cianjur District, Indonesia

**DOI:** 10.14202/vetworld.2023.1736-1746

**Published:** 2023-08-25

**Authors:** Dwida Agustina Suherman, Etih Sudarnika, Trioso Purnawarman

**Affiliations:** 1 Animal Health Training Center of Cinagara, Bogor 16740, Indonesia; 2 Animal Biomedical Sciences Study Program, Postgraduate School, IPB University, Indonesia; 3 Division of Veterinary Public Health and Epidemiology, School of Veterinary Medicine and Biomedical Sciences, IPB University, Bogor 16680, Indonesia

**Keywords:** antibiotic resistance, knowledge, pathways analysis, questionnaire

## Abstract

**Background and Aim:**

Antibiotic resistance is a component of antimicrobial resistance and is often referred to as the silent pandemic. It is one of the causes of global health problems that must be addressed. Resistance occurs due to frequent misuse and overuse of antibiotics by dairy farmers. Therefore, this study aimed to examine the influence of the characteristics of dairy farmers and analyze the variables that directly and indirectly affected the knowledge, attitudes, and practices (KAP) of dairy farmers regarding antibiotic resistance in a dairy farmers’ cooperative in North Cianjur.

**Materials and Methods:**

A cross-sectional design was used with a structured questionnaire validated by *Pearson correlation* and reliability tested with *Alpha Cronbach*. Data were obtained from interviews with 75 dairy farmers in KPSCU, Cianjur District, West Java, Indonesia. The outcome variables were analyzed using descriptive and pathway analyses.

**Results:**

The results showed that dairy farmers had good and sufficient knowledge (42.7%), the attitude level was positive (81.3%), and all farmers had sufficient practices (100%). The results also showed that most dairy farmers were over 40 years of age, with 54.7% having elementary school education. Approximately 50.7% of farmers had been engaged in breeding for 10–20 years, 80% owned their livestock, and 76% had attended training. There was a significant relationship between age, education, training, and knowledge level. Knowledge was the primary factor influencing the overall attitude. In addition, age, education, type of business, knowledge, and attitude were factors that influenced the practice of antibiotic resistance.

**Conclusion:**

Training and education really influenced KAP of farmers. Therefore, the best way to reduce antibiotic resistance is by increasing farmers’ knowledge and understanding of antibiotic resistance and monitoring the use of antibiotics.

## Introduction

Antibiotic resistance is a phenomenon that occurs in livestock and can affect human health through environmental damage or presence of antibiotic residues in animal foods. According to Chokshi *et al*. [1], without appropriate actions taken, antimicrobial resistance (AMR) infections are predicted to cause nearly 10 million deaths annually, with a projected total GDP loss of $100.2 trillion by 2050. This makes antibiotic resistance the leading cause of death globally. In Indonesia, antibiotic use is fairly high and less accurate, increasing the incidence of resistance [2]. The rate of bacterial resistance in Indonesia continues to increase. The previous study by Chandrasekaran *et al*. [3] in India showed increased antibiotic resistance, resulting in increased resistance to *Staphylococcus aureus*, *Escherichia coli*, and methicillin-resistant *S. aureus* bacteria. The AMR Control Committee reported that the prevalence of resistant bacteria increased from 40% to 60% between 2013 and 2019 [4].

Antibiotic resistance in the field is influenced by several environmental factors, such as the ease of obtaining antibiotics, unhygienic environmental conditions, and inadequate sanitation implementation. In many regions globally, antibiotics are usually sold to customers outside the healthcare setting without a medical prescription [5]. Farmers commonly use antibiotics for various purposes, including therapy, prophylaxis, flushing, and growth promotion [6]. A previous study in Lebanon [7] showed that farmers’ practices were directly linked to antimicrobial misuse, including overuse, suboptimal use, and non-compliance with the prescribed duration. Other external factors that influence the occurrence of cultural resistance are closely linked to ingrained habits, while the association of farmers strongly influences the social factor. Internal factors affecting the characteristics of farmers are triggers for antibiotic resistance in the field. This occurrence of antibiotic resistance can be detrimental to farmers, as improper handling of livestock will lead to repeated treatment, prolonged painful conditions, poisoning, weakness, and death in cattle. Approximately 63,000 tons of antimicrobials are globally administered in animal food annually, with projections indicating a 70% increase in livestock by 2030 [8]. The causative factors contributing to resistance include irrational use, lack of supervision from the government, ease of access, and failure to apply proper sanitary hygiene that affects residues of animal food materials. According to O’Neill [9, 10], failure to promptly address antibiotic resistance will lead to 10 million deaths annually. Furthermore, antibiotic residues in food might interact with the human microbiome, promoting the growth of antibiotic-resistant bacteria that can persist in their digestive system for years [11, 12].

One of the efforts made to improve human health is to ensure the safety of food consumed. The health of livestock is crucial in this situation, as it directly impacts the use of antibiotics in the field. According to data from the World Health Organization, antibiotic use increased by 91% globally and 165% in developing countries between 2000 and 2015, making antibiotic resistance one of the world’s ten most dangerous global health threats [13]. Handayanti and Gunawan [14] identified the lack of public knowledge about antibiotic use as the major factor in the occurrence of resistance. An effort to address resistance is the improvement of knowledge, which involves training cattle farmers and animal health officials and disseminating information about antibiotic resistance to the community. In addition, irrational antibiotic use is influenced by various factors, including a lack of knowledge and attitudes toward the overuse of antibiotics and their effect on human health [15]. Cianjur District has the 10^th^ largest cattle population in West Java Province, spanning 2.788 square [16]. To date, no studies have been conducted on antibiotic resistance in cattle farms in Cianjur District, which serves as the basis for this study.

This study aimed to analyze the correlation between the characteristics and KAP of dairy farmers in KPSCU regarding the occurrence of antibiotic resistance. It also aimed to identify the variables that directly and indirectly affected the appearance of AMR in the field, thereby providing benefits related to effective efforts in reducing the occurrence of antibiotic resistance.

## Materials and Methods

### Ethical approval

The Human Research Ethics Committee of Bogor Agricultural University approved this study proposal under the number: 696/IT3.KJV-IPB/SK/2022.

### Study period and location

This study was conducted from July to November 2022 in Dairy Farmers’ Cooperative in North Cianjur (KPSCU), an area with strategic potential for the development and growth of the economy and health. It was held in Cipanas, Pacet, and Sukaresmi Subdistrict of KPSCU, covering 201.09 km^2^. KPSCU is known for having the largest dairy cattle population in Cianjur. This study focused on six dairy cattle farmer groups in KPSCU.

### Study design

This study used a cross-sectional design, incorporating a knowledge, attitudes, and practices (KAP) survey, interviews, and observations of dairy farmers. The variables examined included the characteristics, KAP of farmers in KPSCU. The characteristics included age, type of business, training, education, and duration of business. Farmers’ KAP regarding antibiotic resistance included definition, antibiotic use, the effects of resistance, prevention, control, the application of sanitary hygiene on the farm, and treatment.

### Sample size and population

The sample size was determined based on the population of lactating cattle farmers in KPSCU, which consisted of 126 farms, using WinEpi software (Ignacio de Blas. Facultad de Veterinaria, Universidad de Zaragoza ©2006; http://www.winepi.net),Using WinEpiscope 2.0 software (Ignacio de Blas, Veterinary College, University of Zaragoza© 2006, www.winepi.net), with a confidence level of 95%, a presumption prevalence of 50%, and an error rate of 8%, the calculated sample size was 70 farmers. An additional five farmers were included to increase the response rate, resulted in 75 respondents from six dairy cattle farmer groups in KPSCU. This study was conducted using a cross-sectional design by conducting interviews and observations with these 75 dairy farmers and gathering information on KAP related to antibiotic resistance.

### Data collection

Data were collected using a structured questionnaire through direct interviews. The questionnaire or instrument was tested for validity and reliability to ensure its feasibility before implementation. Before the validation of the questionnaire, a pre-test was conducted with 10 farmers from the study population. The validity of the instrument was assessed using *Product Moment Pearson* correlation, while the reliability was tested using *Alpha Cronbach*. Statements with calculation numbers that yielded a critical value of p < 0.05 were considered statistically significant. The instrument included up to 60 questions describing farmers’ KAP about antibiotic resistance, comprising 20 KAP related questions each.

A questionnaire for assessing farmers’ knowledge was designed with 20 questions regarding resistance to antibiotics. The three answer choices for respondents were true, false, and do not know [17]. A value of 1 was assigned for correct answers, while incorrect or unknown responses were assigned 0 [18]. Questions were divided into positive and negative to reduce the response bias. These positive and negative questions were answered correctly when respondents selected the right and wrong options, respectively. The knowledge was categorized as “good” when respondents’ achieved a score >14, “sufficient” when the score ranged from 7 to 14, and “less” for scores below 7 [19].

The attitude level was assessed using a 2-point Likert scale comprising 20 questions, with response options of agree, disagree, and doubt. The attitude score was assessed by multiplying the answers to the questions by their respective scores. Positive statements were scored as 2, 1, or 0 when respondents selected “agree,” “doubtful/neutral,” or “disagree,” respectively. Meanwhile, for negative statements, the scoring system was reversed. For example, scores of 0, 1, or 2 were assigned when respondents selected “agree,” “doubtful/neutral,” or “disagree,” respectively. The attitude score ranged from 0 to 40 and levels were determined by dividing the differences between maximum and minimum scores. The division results were used as intervals to ascertain the attitude level category [20]. The attitude category was “positive” for scores >27, “neutral” for scores between 14 and 27, and “negative” for scores <14.

The evaluation of farmers’ practices included 20 questions, which were measured using a checklist with statements describing the conditions of the practices carried out. The checklist used a Likert scale with three response options, namely, always, sometimes, and never. The total score for the practice assessment was 60. Good, moderate, and low levels were indicated for scores >47, between 34 and 47, and below 34, respectively. The survey also included 53 questions related to animal health management, hygiene, and sanitation. Some questions and the respondents’ answers are shown in Table-1.

**Table-1:** Some questions and answer respondents about animal care.

Question	Yes	No
If your cow gets sick, do you give antibiotics to your cow?	51	24
Have you ever given a dose of antibiotics more than prescribed?	56	19
Have you ever given a lower dose than prescribed?	42	33
Have you ever stopped giving medication before it was prescribed?	28	47
Apart from the officers and you, is there anyone else giving medication to your cattle?	5	70
Have you ever given human drugs to animals?	21	54

### Statistical analysis

The questionnaires used were validated using the *Pearson correlation* test and their reliability was tested using *Cronbach’s alpha* method. A statement that had a calculation number with a critical value p < 0.05 was considered statistically significant. The data obtained were analyzed with pathway analysis to identify direct and indirect influences and correlations between variables. p < 0.05 was considered statistically significant.

## Results

### Farmers’ practice in antibiotic use

Table-1 shows the answers to several questions aimed at determining the practices carried out by farmers and the causes of antibiotic resistance. As many as 51 farmers stated that antibiotics were administered when cattle were sick, believing in the effectiveness of antibiotics in treating various diseases. During the interview, 56 farmers used high doses for treatment and 42 used low doses. The previous study by Speksnijder *et al*. [21] reported low doses as the primary reason for developing harmful AMR significantly. However, most of the farmers (47) completed the full treatment course. The treatment was typically carried out by officers (70) and most respondents administered herbal medicine independently (69). This result was in contrast with a previous study conducted in Lebanon, where the majority of farmers believed that antimicrobials showed no adverse effects on animals and were more worried about the health of sick cattle and the possibility of recovering without using antibiotics. This situation is strongly linked to traditional practices and knowledge [7]. Furthermore, a small proportion of respondents (21) administered human medicines to the livestock.

### Dairy farmers’ characteristics

Based on the results of the questionnaire interviews regarding education, most of the respondents (41 or 54.7%) were elementary school graduates, followed by high school graduates (22 or 29.3%). In addition, there were 6 (8%) uneducated respondents, 4 (5.3%) college graduates, and 2 (2.7%) high school graduates. In terms of age, the respondents were predominantly adults, with 53.3% being over 40 years old. Among the 75 respondents, 40 were over 40 years old, indicating a mature age group with a greater understanding and experience of various developments. Most of the respondents (60 or 80%) were livestock owners, while the remaining 15 (20%) were part-time workers. Concerning the duration of farming, most (78.7%) had been breeding for more than 10 years, showing their experience in breeding. It was found that 24% had never attended any training, while 76% had attended training in animal health management. However, no farmer had participated in training related to AMR, suggesting a limited knowledge of antibiotic resistance. The characteristic data of dairy farmers in KPSCU are shown in Table-2.

**Table-2:** KPSCU Region dairy farmer’s characteristics.

Characteristic	Frequency	%
Age		
<20 years old	1	1.3
20–40 years old	34	45.3
>40 years old	40	53.3
Type of business		
Owned	60	80
Part worker	15	20
Training		
Yes	57	76
No	18	24
Education		
Non-educated	6	8
Elementary school	41	54.7
Middle school	22	29.3
High school	2	2.7
College	4	5.3
Dairy rising length		
<10 years	16	21.3
10–20 years	38	50.7
>20 years	21	28

### Dairy farmers’ KAP

**Figure-1:**
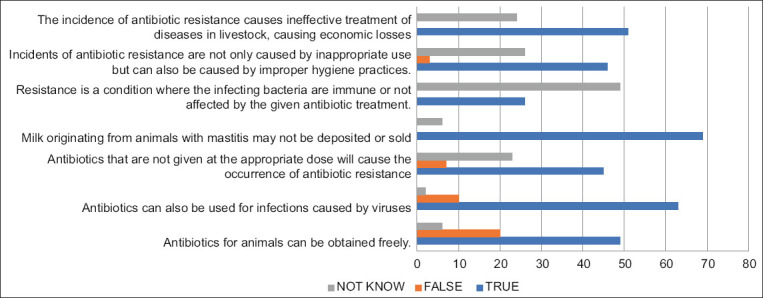
Frequency and percentage of respondents’ knowledge to the answer to some of the 20 questions about antibiotic resistance.

Several responses related to the respondents’ knowledge of antibiotic resistance are shown in Figure-1. Some respondents (49) stated that antibiotics were easily accessible, allowing free usage regardless of the proper dosage and the correct diagnosis. According to Obaidat *et al*. [22], dairy companies in Jordan frequently misused antimicrobials due to the ease of access, allowing farmers to prescribe antibiotics independently for their livestock. A total of 45 respondents acknowledged that inappropriate drug doses could lead to antibiotic resistance, while some respondents still lacked this knowledge. Furthermore, seven respondents stated that inappropriate dosing did not contribute to antibiotic resistance. As many as 69 respondents knew that milk from cattle with mastitis could not be sold or stored. This result is consistent with a study by Oliver and Murinda [23], which suggests that intramammary use of antibiotics in dairy cattle could contribute to the increase in pathogen-resistant antimicrobials found in milk. There were still several respondents who used antibiotics for other diseases. Most respondents (63) believed in the effectiveness of antibiotics in treating diseases caused by viral infections. This misconception may be attributed to a lack of understanding about antibiotic resistance among some respondents (49). This is in contrast to the results of a study in Lebanon stating that most of the respondents believed that antibiotics should be used only for the treatment of infections [7].

**Figure-2:**
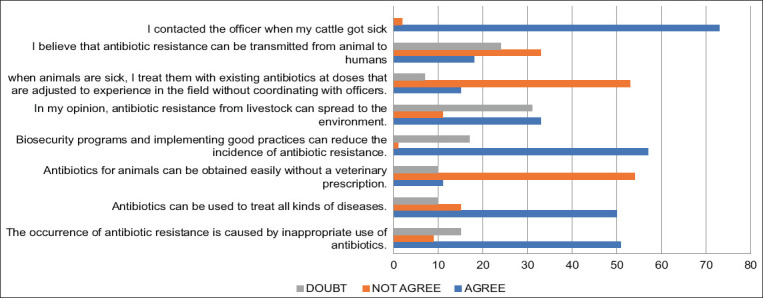
Frequency of respondents to some questions about attitudes toward antibiotic resistance.

Figure-2 shows the responses to several questions regarding schematic action. Most respondents (73) expressed the intention to contact KPSCU staff when the animals were sick. A total of 57 respondents acknowledged the role of biosecurity and good practices in reducing the incidence of antibiotic resistance. In terms of attitude, 15 respondents indicated treating the animals based on experience without consulting animal health officials or veterinarians, while 53 of 75 disagreed. A majority (50) stated that antibiotics could be used to treat all diseases, irrespective of appropriateness, leading to antibiotic resistance. Only a few respondents knew the potential of antibiotic resistance transmission from animals to humans. The previous studies by Tepeli and Zorba [24], and CDC [25] explains that outbreaks of resistance were associated with contaminated food and contact with livestock, pets, and pet food.

The level of farmers’ KAP related to the management of antibiotic resistance was assessed by assigning a score to each variable, with the frequency being the number of farmers. The majority of respondents were categorized as having sufficient practice (100%) and a positive attitude (81.3%), while a few had poor knowledge (14.7%). Table-3 shows the categories of KAP of farmers toward antibiotic resistance. The knowledge of respondents was categorized as good (32) and moderate (32). This can be attributed to experience in livestock management, participation in training on livestock health, and the availability of annual member meetings for sharing problems and solutions related to livestock. For the attitude category, 61 respondents showed a positive attitude, which was influenced by the knowledge possessed and played a role in the decision-making process. Attitude greatly influenced the practice of respondents, but all practices were categorized as moderate. This is because, although most respondents had positive attitudes, they still did not apply ideal practices. The lack of understanding or unawareness of the adverse effects of administering incorrect drugs and the sense of being correct contributed to this situation. The common thread for reducing the incidence of resistance lies in the application of improved practices through positive farmers’ attitudes by increasing knowledge and insight into efforts to control antibiotic resistance through training, social media outreach, meetings with members, and the participation of the government and related agencies to help increase the understanding of farmers and promote better practices.

**Table-3:** KAP assessment of dairy farmers in KPSCU.

Characteristics	Frequency	Percentage
Knowledge		
Good	32	42.7
Moderate	32	42.7
Poor	11	14.7
Attitude		
Positive	61	81.3
Neutral	14	18.7
Negative	0	0
Practice		
Good	0	0
Moderate	75	100
Poor	0	0

KAP=Knowledge, attitudes, and practices

### The relationship between personality and KAP

The results of the *Pearson correlation* test are shown in Table-4, indicating that the level of knowledge among dairy cattle farmers correlates with training, age, and attitude (p ≤ 0.01). Farmers’ attitude was positively correlated with training, knowledge, and practice (p ≤ 0.01). In terms of practice, there was a negative correlation between the type of business owner and practice, and a positive correlation with attitudes, at a significance level of p ≤ 0.01. This suggests that the type of businesses owned by breeders increases the tendency to have bad practices. There is a possibility that breeders do not focus on one business, resulting in less than ideal practices. To improve this situation, efforts should be made to increase farmers’ understanding of good livestock practices.

**Table-4:** Pearson correlation analysis of dairy farmer’s characteristics, knowledge, attitudes, and practices regarding antibiotic resistance in KPSCU.

Correlations	A	B	C	D	E	F	G	H
Training (A)								
Pearson correlation	1	−0.125	0.454**	0.357**	0.181	−0.024	0.097	0.080
Sig.		0.286	0.000	0.002	0.120	0.838	0.406	0.493
n	75	75	75	75	75	75	75	75
TOB (B)								
Pearson correlation	−0.125	1	−0.194	−0.126	−0.424**	−0.053	−0.114	−0.334**
Sig.	0.286		0.095	0.281	0.000	0.652	0.329	0.003
n	75	75	75	75	75	75	75	75
Knowledge (C)								
Pearson correlation	0.454**	−0.194	1	0.622**	0.160	0.224	0.338**	0.072
Sig.	0.000	0.095		0.000	0.169	0.053	0.003	0.538
n	75	75	75	75	75	75	75	75
Attitudes (D)								
Pearson correlation	0.357**	−0.126	0.622**	1	0.331**	0.099	0.109	0.000
Sig.	0.002	0.281	0.000		0.004	0.400	0.351	1.000
n	75	75	75	75	75	75	75	75
Practice (E)								
Pearson correlation	0.181	−0.424**	0.160	0.331**	1	0.214	0.165	0.051
Sig.	0.120	0.000	0.169	0.004		0.066	0.158	0.665
n	75	75	75	75	75	75	75	75
Education (F)								
Pearson Correlation	−0.024	−0.053	0.224	0.099	0.214	1	−0.018	−0.003
Sig	0.838	0.652	0.053	0.400	0.066		0.875	0.980
n	75	75	75	75	75	75	75	75
Age (G)								
Pearson correlation	0.097	−0.114	0.338**	0.109	0.165	−0.018	1	0.305**
Sig.	0.406	0.329	0.003	0.351	0.158	0.875		0.008
n	75	75	75	75	75	75	75	75
Dairy rising l ength (H)								
Pearson correlation	0.080	−0.334**	0.072	0.000	0.051	−0.003	0.305**	1
Sig.	0.493	0.003	0.538	1.000	0.665	0.980	0.008	
n	75	75	75	75	75	75	75	75

** Correlation is significant at the 0.01 level (2-tailed). *Correlation is significant at the 0.05 level (2-tailed). Sig.=Significant

**Figure-3:**
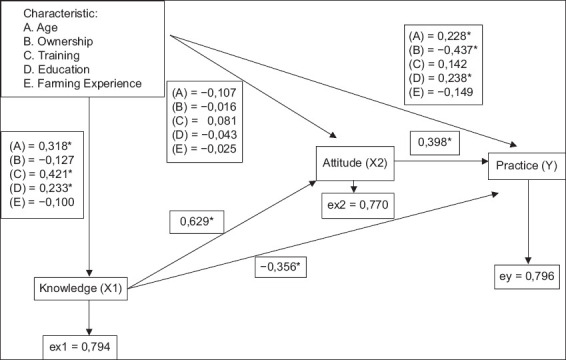
Value of path coefficients for research variables of knowledge, attitudes, and practices of dairy farmers on controlling antibiotic resistance in KPSCU region. ex1=Residual, ex2=Residual x2 dan, ey=residual y, *Significant effect.

The pathway coefficient values indicate the correlation between variables according to the study’s conceptual framework. The results of the path analysis and the magnitude of direct and indirect influences of each independent and dependent variable are shown in Figure-3. Age, education, and training are characteristics of farmers that directly influence the level of knowledge, with path coefficients (r) of 0.318, 0.421, and 0.233, respectively. There were no direct effects of farmers’ characteristics on attitudes. However, the level of attitudes was directly and significantly influenced by the level of knowledge, with r value of 0.629. Furthermore, age, ownership, education, attitudes, and knowledge directly affected the practice level, with r values of 0.228, −0.437, 0.238, 0.398, and −0.356, respectively.

### Individual characteristics and knowledge correlation

The direct influence of individual characteristics on dairy farmers’ knowledge in KPSCU is indicated by r (Table-5). The effect of the training on farmers’ knowledge about antibiotic resistance had the highest percentage when compared to other characteristic variables, with a total effect of 20.79% of 36.79%. Training is an informal education that can enhance farmers’ knowledge of important aspects of breeding. This illustrates that breeders who have participated in the training will be knowledgeable about antibiotic resistance. Other farmers’ characteristics that affect knowledge related to antibiotic resistance are age and education. Age had a total effect of 15.70% of 36.79%, also indicating a significant influence. Older farmers tend to be more mature when dealing with incidents on the farm due to knowledge gained from past experiences. Education had a total influence of 11.50% of 36.8%, indicating a significant influence on farmers’ knowledge. Farmers with higher formal education had better knowledge and understanding of controlling the incidence of antibiotic resistance.

**Table-5:** Direct and indirect effects, as well as the significance of variables influencing farmer knowledge of antibiotic resistance in KPSCU.

Variable Effect	Direct Effect	Total Effect	Percentage	Significant
a. toward X1	0.318	0.318	15.70	0.002*
b. toward X1	−0.127	−0.127	−6.27	0.220
c. toward X1	0.421	0.421	20.79	0.000*
d. toward X1	0.233	0.233	11.50	0.018*
e. toward X1	−0.100	−0.100	−4.93	0.348
Total	0.745			
	(36.80%)		36.8	

a=Age, b=Type of business, c=Training, d=Education, e=Dairy raising length. X1=Knowledge, X2=Attitude, Y=Practice.

* Indicates a significant correlation α=0.05; confidence interval 95%

### Individual characteristics, attitude, and knowledge correlation

Table-6 shows that attitudes are directly and indirectly influenced by characteristics and knowledge according to the study’s conceptual framework. The knowledge variable had the highest influence on the attitude of farmers toward antibiotic resistance compared with other characteristic variables. The total effect of the knowledge variable on attitude was 25.84% of 40.6%, or about 63.65% was influenced by knowledge. Furthermore, persons’ attitude is formed based on the knowledge they possess. A significant difference in attitude suggests that farmers with greater knowledge are more capable of determining attitude, acquired through learning or based on experience. Attitude is essential in determining the success of controlling antibiotic resistance practices in livestock.

**Table-6:** The direct and indirect effects, as well as the significance of variables influencing farmer’s attitudes toward antibiotic resistance in KPSCU.

Variable effect	Direct effect	Indirect effect through X1	Total effect	%	Significant
a toward X2	−0.107	0.200	0.093	3.82	0.314
b toward X2	−0.016	−0.079	−0.095	−3.90	0.871
c toward X2	0.081	0.264	0.345	14.17	0.452
d toward X2	−0.043	0.146	0.103	4.23	0.658
e toward X2	−0.025	−0.062	−0.087	−3.57	0.811
X1 toward X2	0.629	-	0.629	25.84	0.000*
Total	0.519	0.469			
	(21.3%)	(19.3%)		40.6	

a=Age, b=Type of business, c=Training, d=Education, e=Dairy raising length. X1=Knowledge, X2=Attitude, Y=Practice.

* Indicates a significant correlation α=0.05; confidence interval 95%

### Individual characteristics, KAP correlation

Table-7 shows that practice is directly and indirectly influenced by individual characteristics, knowledge, and attitudes. The r represents the direct and indirect influence of individual characteristics, knowledge, and attitudes on the practice of dairy farmers related to the control of antibiotic resistance in KPSCU. Variables that significantly influence the practice of dairy farmers are the type of business, attitude, age, education, and knowledge. Type of business had the highest percentage, indicating a negative correlation (−37.6%) and an indirect effect on practice. This was because the indirect effect (0.007) value was greater than the direct effect (−0.437). The type of livestock business did not guarantee good practices and it had an indirect influence on control of antibiotic resistance. The attitude variable significantly influenced practice, accounting for 34.7% of 36.6%, and directly affected farmers’ practice. The results of the study showed a very influential attitude towards antibiotic resistance control practices, so it can be concluded that the better attitude of dairy farmers, the more effective their practice of controlling antibiotic resistance. This was consistent with the study by Noviana *et al*. [26], where a positive attitude among livestock owners contributed to better disease control practices. Age is another variable that influenced practice, with a percentage of 33.1% of 36.6% and a positive correlation that directly and indirectly influenced practice. Older respondents tend to exhibit more positive attitudes, potentially due to their experience and understanding of animal health management. This helps to control antibiotic resistance by applying ideal practices based on their experience and understanding. Formal education is the next variable with a significant effect on practice, with a percentage of 17.1% of 36.6%. This shows that the higher the education of a breeder, the better the practice of controlling antibiotic resistance. This is in line with a previous study by Kustiningsih *et al*. [27] that highlighted the importance of educational level as a positive factor in the implementation of brucellosis prevention practices. The final variable that significantly influenced practice was knowledge (−9.2%), showing a negative correlation and an indirect effect through attitude. This shows that knowledge indirectly influences practice through attitudes. Farmers with higher knowledge tend to exhibit worse practices, attributed to a lack of proper understanding regarding antibiotic resistance. Some farmers still believe that resistance is not transmitted through the food chain or does not have a real impact on their livestock. This may be caused by their knowledge from workshops, training, or counseling that is unrelated to antibiotic resistance. The path analysis obtained shows that good practice requires a positive attitude and extensive knowledge of controlling antibiotic resistance. The efforts to enhance knowledge can be made by holding trainings, counseling for farmers and officers, and conducting outreach through social media.

**Table-7:** The direct and indirect effects, as well as the importance of variables influencing farmer’s practices in the control antibiotic resistance in KPSCU.

Variable effect	Effect				Indirect total	Total effect	%	p-value

	Direct	Indirect						

		X1	X2	X1*X2				
a toward Y	0.228*	0.113	−0.042	0.080	0.151	0.379	33.1	0.043*
b toward Y	−0.437*	0.045	−0.006	−0.032	0.007	−0.430	−37.6	0.000*
c toward Y	0.142	−0.150	0.032	0.105	−0.013	0.129	11.3	0.209
d toward Y	0.238*	−0.083	−0.017	0.058	−0.042	0.196	17.1	0.022*
e toward Y	−0.149	0.036	−0.009	−0.025	0.002	−0.147	−12.8	0.173
X1 toward Y	−0.356*	-	0.250	-	0.250	−0.106	−9.2	0.017*
X2 toward Y	0.398*	-	-	-	-	0.398	34.7	0.002*
Total	0.064	−0.039	0.208	0.186	0.355	0.419		
	(5.59%)	(−3.40%)	(18.17%)	(16.24%)	(31.01%)		36.6	

a=Age, b=Type of business, c=Training, d=Education, e=Dairy raising length. X1=Knowledge, X2=Attitude, Y=Practice.

* Indicates a significant correlation α=0.05; confidence interval 95%

## Discussion

The majority of respondents were over the age of 40 (53.3%), indicating a mature group with significant life experiences. However, most farmers had a low degree of formal education, with most being primary school graduates (54.7%). This suggests that they may require guidance and education regarding appropriate actions related to antibiotic use. Individuals with higher education levels tend to acquire knowledge more easily. This aligned with the results of Gyekye and Salminen [28], where higher education levels influenced an individual’s performance to become better and more directed. Education enhances knowledge and produces a better attitude [20]. About 78.7% of farmers had been raising dairy cattle for more than 10 years, indicating their extensive history in the field. The majority of farmers (80%) own cattle as their primary business and most (76%) attended animal health training, indicating a certain level of animal knowledge. Improved knowledge can be achieved through education related to proper antibiotic use, which also serves to correct the misperceptions about antibiotic use in society [29]. According to El Sherbiny *et al*. [30], increasing public awareness about the proper use of antibiotics, promoting the attitude, and changing the misbehavior towards antibiotics. With this intention campaigns, postings, and slogans could be used to inspire people about using antibiotics when indicated and prescribed only by the health professional for protection from their negative consequences [30], interventions, modules, and group discussions enhanced the knowledge, attitudes, and behaviors related to the rational use of antibiotics. In 2018, community-based health education programs in any sector of the community were identified as the most effective way of directing the public toward the rational use of antibiotics [31]. In this study, the most significant influence on knowledge was training, education, and age. The correlation between knowledge and training in this study is in line with Niati *et al*. [32], who argued that training is a process of transferring particular knowledge, skills, and attitudes to increase people’s knowledge and capability to carry out their jobs more effectively and in compliance with standards. It is essential to increase knowledge and insights related to the control of antibiotic resistance in the field. This can be achieved through training programs and improved dairy farmer education. Knowledge rises with education and is consistent with the findings of this study, which shows a strong relationship between knowledge and education. Efforts to enhance knowledge can be made through introductions to farmers or through education and training. Another variable with a clear influence on knowledge was age, suggesting that farmers over the age of 40 had more knowledge about livestock management. These results aligned with a previous study by Budiman [33], which observed an increase in knowledge with age. In addition, education also had a significant impact on knowledge. The level of education played a role in determining how easily individuals receive information and increase their knowledge [34]. The experience of farmers, which is closely related to age, influenced the knowledge possessed. Farmers with a high level of education tend to accept new things more easily. This is in line with the results of this study that there is a significant correlation between education and knowledge [35]. Based on this result, training activities aimed at improving farmers’ knowledge and skills in breeding livestock were directly compared to the existing knowledge. An individual’s knowledge is influenced by their background, such as their age, marital status, education, and social environment (residence and work environment). Knowledge can change and develop according to abilities, needs, experiences, and level of reception of information present in the environment [36]. This was similar to the results of Dankar *et al*. [7], which emphasized the importance of implementing training and awareness programs, as well as establishing policies and regulations to reduce antibiotic use and hinder the spread of AMR in Lebanon.

Regarding KAP information obtained, farmers’ knowledge levels were categorized as moderate (42.7%) and good (42.7%). The majority of farmers showed positive attitudes (81.3%), while a smaller proportion (18.7%) showed neutral attitudes. Positive attitudes are influenced by knowledge and thought toward a particular issue [20]. According to the results obtained from farmers’ attitudes toward the application of sanitary hygiene and animal health management, it was observed that the attitude was good. However, it is necessary to apply the understanding related to the proper and wise use of medicines. In the practice category, all respondents were in the moderate category (100%) and this suggested a lack of knowledge about antibiotic resistance, leading to suboptimal practices.

In this study, knowledge had a significant influence on attitude and it serves as the basis of a person’s attitude [34]. Positive attitudes can be shaped by good knowledge, thoughts, beliefs, and emotions [37]. Farmers with a good level of knowledge are more likely to develop a good mindset, belief, and emotional outlook, which increase the probability of taking appropriate actions [9]. In addition, knowledge influences a person’s actions [38]. As knowledge grows, individuals gain a better understanding of the situations they encounter, leading to positive attitudes, particularly among farmers. In Table-6, the direct influence of characteristics and knowledge on attitudes is more significant than the indirect influence, indicating that these factors have a substantial influence on attitudes.

The overall impact of several factors on practice was 36.6%, as described in Table-7. These factors include age, type of business, training, education, length, attitude, and knowledge. Based on the results of the path analysis, the type of business, attitude, knowledge, and age significantly influenced the occurrence of antibiotic resistance. The direct influence of the type of business on practice had a negative correlation with r value of –0.437 and the indirect influence had a positive correlation with r value of 0.007. The indirect influence was greater compared to direct influence; hence, indirect types of business have a significant influence on practice. These results imply that farmers who own their businesses tend to have poorer practices than those who work for others. This may be attributed to their limited knowledge of dairy farming, as it is not their primary enterprise. In this study, part-time farmers showed better coping strategies when their livestock fell ill, opting for herbal remedies instead of commercial drugs. This indicated that part-time farmers had better knowledge about animal health management, enabling the management of sick animals without antibiotics. These results align with Mboe *et al*. [39] that the more knowledge a person has, the more positive a person’s attitude is, and the better the practices.

Attitudes have a significant effect on practice (94.8%), with r value of 0.398. Farmers with exemplary attitudes will implement good practices based on their knowledge. Education and knowledge also have a tangible impact on practice and the experience a farmer has will also impact practice. This result was reinforced by a statement by Budisuari *et al*. [40] that education is a factor that can influence actions and correct practices. Attitude affects the practice of what is learned and known [27]. Respondents’ attitude toward antibiotic use requires improvement as some continue to use antibiotics for all diseases and improper dosages for treatment. In a survey conducted by Geta and Kibret [41], farm owners/workers stated that 72.5% of farmers obtained antibiotics without prescriptions. Antibiotics were used hoping that the cattle would recover quickly and not increase their expenses. Agricultural policies and milk prices also shape the way farmers practice and perspective, as observed in previous studies by Fischer *et al*. [42].

Knowledge had a negative coefficient value on practice, with r value of −0.356, indicating an opposite correlation or direction of influence. The practice of antibiotic resistance decreases as farmers’ understanding increases. This can be attributed to suboptimal knowledge among farmers, including beliefs in the lack of evidence regarding antibiotic resistance transmission to humans or the perception that antibiotics function as growth promoters. Some farmers lack the understanding that sick cattle should be given antibiotics only after establishing the cause of the disease, leading to poor control of antibiotic resistance. The knowledge that these farmers have is not in line with the ideal practices to be applied to control antibiotic resistance. This contradicted the statement by Noviana *et al*. [26], indicating a positive direct impact of knowledge on practice, with higher knowledge levels corresponding to better practice.

Formal education directly and significantly affected practice (p < 0.05), with r value of 0.238. This indicates that the better the education of farmers, the higher is the level of practice to control antibiotic resistance. The results of path analysis are supported by Wicaksono *et al*. [20], who stated that respondents with higher education are more receptive to new interventions related to biosecurity practices. The education of dairy farmers can be developed through education training activities or workshops related to antibiotic resistance. Efforts are required to increase farmers’ knowledge and education and training can achieve this [34].

The age variable directly and significantly affected practice with a positive correlation (90.4%) and r value of 0.228. This suggests that the older respondents have a more positive reaction to antibiotic resistance, resulting in better practice. Furthermore, with increasing age, there is an enhanced understanding and knowledge acquired through experience. These findings are in line with El-Sokkary *et al*. [43], where older, more experienced, and trained prescribers showed proper practice. However, the results of a previous study by Kustiningsih *et al*. [27], which found a negative correlation between age and attitude, were contraindicated. This suggests that age alone does not guarantee better actions and attitudes toward issues, as younger people are more receptive to new knowledge.

This study provides valuable insights from the field, serving as a crucial foundation for the government in determining the proper strategic steps. It can be used as a reference for conducting training programs for farmers and animal health officers regarding the control of antibiotic resistance. The limitations of this study include the refusal of some farmers to be interviewed due to the presence of foot and mouth disease outbreaks in the field, which prevented the collection of population data.

Based on the results of this study, there should be an improvement in factors that significantly influence farmers in controlling antibiotic resistance, such as farmers’ knowledge. Practical implications include training farmers and officers as well as implementing communication, information, and education programs through social media or other dissemination channels. All parties must play a role in the management of antibiotic resistance incidents. The government can use these results to regulate the use of antibiotics in the field, supervise the antibiotics used, and guide farmers in choosing appropriate treatments for their farms.

## Conclusion

Attitude had the most significant direct effect on farmers’ knowledge of the incidence of antibiotic resistance. Attitude was significantly influenced by farmers’ knowledge and training had the most significant effect on knowledge. Control efforts could be carried out by conducting training to increase the knowledge and understanding of farmers regarding antibiotic resistance; hence, these incidents would be reduced.

## Authors’ Contributions

DAS, ES, and TP: Conceived the idea, designed the study, developed the theory, and prepared the tools and materials. DAS: Collected the data. DAS and ES: Analyzed the data and wrote the manuscript with input from all authors. All authors participated in the discussion of the results and contributed to the final manuscript, provided critical feedback, and helped shape the study, analysis, and manuscript. All authors also have read, reviewed, and approved the final manuscript.
